# Age-Related Differences in Pediatric Burn Characteristics: A Retrospective Analysis at Cabell Huntington Hospital

**DOI:** 10.7759/cureus.80019

**Published:** 2025-03-04

**Authors:** Armein Rahimpour, Isabella G Stuart, Nathan Fox, Kelsie Roberts, Thomas Cassier, Karim Abdelgaber, Andrew Weaver, Curtis W Harrison, Paul Bown, Rahman Barry

**Affiliations:** 1 General Surgery, Marshall University Joan C. Edwards School of Medicine, Huntington, USA; 2 Trauma and Surgical Critical Care, Marshall University Joan C. Edwards School of Medicine, Huntington, USA; 3 Plastic and Reconstructive Surgery, Marshall University Joan C. Edwards School of Medicine, Huntington, USA

**Keywords:** age-related differences, burn injury outcomes, burn prevention strategies, burn sources, flame burns, pediatric burn injuries, resource allocation, scald burns, socioeconomic disparities

## Abstract

Background: Pediatric burn injuries are often unintentional and associated with significant morbidity and mortality. In Appalachia, pediatric burn management faces many challenges such as geographic isolation from specialized burn units. Although it is important to lower the incidence of unintentional burn injuries in the pediatric population, there is a lack of research that focuses on differences among age groups in the region of Appalachia. Our study aims to identify factors impacting different age groups in the pediatric population and understand which group is at a higher risk.

Methods: This retrospective study included 218 pediatric patients aged 0-18 years admitted between January 2010 and June 2023. Patients were stratified into four age groups (0-5, 6-10, 11-15, and 16-18 years). Data on gender, burn sources, length of stay (LOS), total body surface area (TBSA) affected, body mass index (BMI), and inhalation injuries were analyzed. Statistical tests included chi-squared tests for categorical variables and analysis of variance (ANOVA) for continuous variables, with significance set at p<0.05.

Results: The study cohort consisted of 218 pediatric patients aged 0-18 years, consisting of 130 (56%) males with an average patient age of 6.9 years (SD ± 6.2). The cohort was further divided into four groups: 0-5 years (109, 47%); 6-10 years (37, 16%); 11-15 years (37, 16%), and 16-18 years (35, 15%), with significant difference in distribution of patients across (p<0.0001). Scald burns were most common in the 0-5-year group (80%) and 6-10-year group (75%), while flame burns were predominant in the 11-15-year group (60%) and 16-18-year group (65%). Significant variability was also noted in LOS (p=0.0017), TBSA (p=0.0112), and BMI (p=0.0003). The average LOS was 2.42 days (SD ± 3.7) in the 0-5-year group, 3.24 days (SD ± 4.1) in the 6-10-year group, 3.41 days (SD ± 4.8) in the 11-15-year group, and 5.8 days (SD ± 5.2) in the 16-18-year group. The average TBSA was 4.36% (SD ± 7.3) in the 0-5-year group, 5.16% (SD ± 8.1) in the 6-10-year group, 8.51% (SD ± 12.6) in the 11-15-year group, and 6.17% (SD ± 8.9) in the 16-18-year group. The average BMI was 19.56 (SD ± 2.3) in the 0-5-year group, 20.81 (SD ± 3.1) in the 6-10-year group, 24.11 (SD ± 3.8) in the 11-15-year group, and 25.86 (SD ± 4.2) in the 16-18-year group.

Conclusions: Distinct age-related patterns were observed in a number of burn patients, including burn source, LOS, TBSA, and BMI. Younger children sustained primarily scald burns with shorter hospital stays and lower TBSA, while adolescents experienced more severe flame burns and longer hospital stays with higher TBSA. These findings emphasize the need for age-specific prevention programs and resource allocation, particularly for older children facing greater burn severity. Further research should focus on long-term outcomes and refining prevention strategies.

## Introduction

Pediatric burn injuries are often unintentional and come with their own unique challenges in addition to posing as a global health concern. The World Health Organization (WHO) reports that over 180,000 deaths per year in burn-related injuries [1]. Although the incidence of massive burns is on the decline, burns that remain serious enough are still complicated clinical problems, providing the reason for the increase in burned children concentrated in regional centers [2]. These injuries vary by age due to developmental, environmental, and behavioral factors, such as young children’s increased exposure to scalding liquids and adolescents’ susceptibility to risk-taking behaviors [2-3].

The Appalachian regions, which extend across approximately 13 states, are notorious for their low-density populated areas, low economic activity, and significantly higher rates of poverty which explains why this region has difficulty in addressing burn injuries [3]. In Appalachia, pediatric burn incidences have their own setbacks regarding the challenges faced, including geographic isolation from specialized burn units and overall higher rates of comorbidities [4-5]. Although it is important to lower the incidence of unintentional burn injuries in the pediatric population, there is a lack of research that focuses on differences among age groups in the region of Appalachia.

Current studies highlight the differences in the occurrence and causes of pediatric burn injuries, with the risk profile changing among different age groups [6]. The younger children group is more prone to scalds involving hot liquids that are predominately preventable and happen in a home environment, while the older children group experience burns dealing with flames, reflecting developmental changes and possible cultural and socio-economic differences [1,7-8]. There has been no effort in research to try and explain the relationship between disparities communities face and burn outcomes, particularly in disadvantaged and underserved areas like Appalachia. The increased prevalence of obesity and socio-economic hardship in this region further complicates burn recovery and brings to light the importance of research focused on Appalachia and potential risk factors [9].

Our study aims to identify this lack of research by examining how the source of burn, scald, or flame burn, for example, and overall severity, measured by length of stay (LOS) and total body surface area (TBSA), differ among the grouped pediatric patients (0-18 years) at Cabell Huntington Hospital in Huntington, West Virginia. Cabell Huntington Hospital is the only facility that’s equipped with burn intensive care unit (BICU) services for the Appalachian region which is why geographic isolation among certain groups fails to receive care. Due to children’s natural curiosity and poor understanding of risks and potential hazards, this population is uniquely predisposed to burn injuries, rendering them vulnerable and emphasizing the need for a burn unit to meet their needs [1]. By analyzing factors such as burn type, TBSA, BMI, and LOS, this research aims to understand the patterns and challenges associated with pediatric burns within Appalachia. Our research has the potential to enhance outcomes of pediatric burn patients in poorly served regions and to deliver better burn care despite region-specific disparities [10]. By investigating these variables among pediatric burn victims, this research can provide knowledge into ways to benefit and improve the prognosis of pediatric burn victims in Appalachia.

## Materials and methods

Study design and population

This retrospective study analyzed data from 218 patients aged 18 years and younger admitted for burn injuries between January 1, 2017, and January 1, 2023. The study was approved by the Institutional Review Board (approval number: 2063568-1) at Marshall University. Patient records were reviewed retrospectively from the registry at Cabell Huntington Hospital’s BICU. This hospital is an academic teaching hospital, a regional referral center, and an American College of Surgeons-verified Level-2 Trauma Center located in Huntington, West Virginia. The dataset was curated through a formal request to the hospital’s Information Technology (IT) department.

Inclusion and exclusion criteria

The study included all patients aged 18 years and younger admitted with burn injuries during the study period. Patients with non-burn conditions such as Stevens-Johnson syndrome, road rash, frostbite, or other unrelated injuries were excluded after manual chart review. A total of 218 patients were included in the final analysis. A retrospective analysis was conducted to evaluate the distribution of pediatric burn cases and their association with age groups (0-5, 6-10, 11-15, and 16-18 years) at Cabell Huntington Hospital. Data from 218 burn cases were analyzed to explore the distribution of cases by age group and to investigate factors potentially associated with age: gender, LOS, inhalation injury, source of burn, BMI, and TBSA. A Chi-square test was used to compare observed and expected burn case counts across age groups for categorical groups. A one-way analysis of variance (ANOVA) assessed differences in mean across age groups for continuous variables. Statistical significance was defined as p<0.05. All statistical analyses were performed using R version 4.2.0 and Python version 3.11. Data visualization was performed using the Matplotlib and Seaborn libraries.

## Results

Table 1 shows the descriptive statistics for the sample. The study consisted of 218 pediatric patients aged 0-18 years, consisting of 96 (44%) females and 122 (56%) males. The average patient age in this study was 6.9 years (SD ± 6.2). This was further divided into four age groups: 0-5 years (109 patients, 50%), 6-10 years (37 patients, 17%), 11-15 years (37 patients, 17%), and 16-18 years (35 patients, 16%). A chi-squared test revealed significant differences in the distribution of patients across these age groups (χ^2^=30.14, p=1.29×10^−6^). The distribution of burn sources was analyzed using a chi-squared test, which revealed a significant difference among age groups (χ^2^=55.42,p=1.52×10^−7^). Scald burns were most common in the 0-5-year group (80%) and 6-10-year group (75%), while flame burns were predominant in the 11-15-year group (60%) and 16-18-year group (65%). Differences in LOS between age groups were assessed using a one-way ANOVA. The test indicated significant variability (F=5.22, p=0.0017). The average LOS was 2.42 days (SD ± 3.7) in the 0-5-year group, 3.24 days (SD ± 4.1) in the 6-10-year group, 3.41 days (SD ± 4.8) in the 11-15-year group, and 5.8 days (SD ± 5.2) in the 16-18-year group. A one-way ANOVA for TBSA showed significant differences among age groups (F=3.79, p=0.0112). The average TBSA was 4.36% (SD ± 7.3) in the 0-5-year group, 5.16% (SD ± 8.1) in the 6-10-year group, 8.51% (SD ± 12.6) in the 11-15-year group, and 6.17% (SD ± 8.9) in the 16-18-year group. BMI differences were evaluated using a one-way ANOVA, revealing significant variability across age groups (F=6.50, p=0.0003). The average BMI was 19.56 (SD ± 2.3) in the 0-5-year group, 20.81 (SD ± 3.1) in the 6-10-year group, 24.11 (SD ± 3.8) in the 11-15-year group, and 25.86 (SD ± 4.2) in the 16-18-year group. The presence of inhalation injuries was recorded. No inhalation injuries were recorded in the 0-5- and 6-10-year groups. The 11-15-year group had two cases, and the 16-18-year group had one case.

**Table 1:** Pediatric Overall Burn Injury Characteristics and Statistical Analysis THD: total hospital days; days: the average number of days patients were hospitalized; TBSA: total body surface area burned; %: the percentage of body surface area affected by burns; BMI: body mass index, kg/m²); SD: standard deviation Inhalation Injury (count, %), Age (years), p-value: Statistical significance comparing age groups; values <0.05 indicate significant differences. The chi-square test was used to analyze the categorical groups and ANOVA for continuous values.

Variable	Number (%)	Age (Mean ± SD)	LOS (Mean ± SD)	TBSA (Mean ± SD)	BMI (Mean ± SD)	Inhalation Injury (count, %)
All Ages	218 (100)	6.88 ± 6.20	3.14 ± 4.43	5.53 ± 6.74	21.41 ± 8.43	3 (1.29%)
0-5	109 (50)	1.98 ± 1.15	2.42 ± 3.77	4.36 ± 4.62	19.56 ± 9.52	0 (0.00%)
6-10	37 (17)	7.68 ± 1.36	3.24 ± 3.01	5.16 ± 5.26	20.81 ± 5.57	0 (0.00%)
11-15	37 (17)	13.24 ± 1.30	3.41 ± 4.28	8.51 ± 11.37	24.11 ± 7.96	2 (5.41%)
16-18	35 (16)	17.29 ± 0.83	5.80 ± 6.91	6.17 ± 6.46	25.86 ± 6.77	1 (2.86%)
p-value	1.12 × 10⁻¹⁵	0.000001	0.001699	0.011186	0.000317	1

## Discussion

Our study examined the differences among 218 pediatric burn patients who received care at Cabell Huntington Hospital in Huntington, the sole BICU in West Virginia. The pediatric patients were stratified according to four groups, “age 0-5”, “age 6-10”, “age 11-15”, and “age 16-18.” The mean age for the pediatric population in this study was 6.88 years. Compared to concurrent research with average ages of 5 and 5.46 years, our higher mean age could be from our smaller sample size compared to much larger patient populations [11-12]. Regional variations in Appalachia based on living conditions and safety measures could also be a variable. Socio-economic factors also play a role due to older children taking part in more risky behaviors, increasing their susceptibility to burns.

The results from Table 1 highlight the differences among each age group and variable factors that potentially determine burn injury in pediatric populations from our region. Our results illustrate the majority of scald burns happened in the younger children groups (0-10 years), and flame burns happened in the older children groups (11-18 years) which is consistent with previous research studies [1,7]. The younger children’s higher incidence of scald injuries suggests increased exposure to hot fluids within the household environment, while older children are more susceptible to flame burns due to their innate risky behavior and greater exposure to hazardous situations [1].

The presence of inhalation injuries was documented, though it was found to have no significant impact on pediatric burns in our population, mainly due to low incidents. Inhalation injuries are commonly associated with burn injuries, particularly flame-related burns, and are known to increase mortality. However, this trend was not observed in our results [13]. No cases of inhalation injury were recorded in the 0-5 and 6-10-year age groups. In contrast, two cases were noted in the 11-15-year group, and one case in the 16-18-year group, totaling three cases among older children. The presence of inhalation injuries in this age group may be attributed to behavioral factors such as risk-taking tendencies or greater exposure to fire and smoke at the time of injury.

These findings underscore the importance of targeted prevention strategies tailored to different age groups. For younger children, efforts should focus on reducing preventable scald injuries at home, while fire safety education and risk-reduction measures are crucial for older children to minimize the overall incidence of pediatric burns [6].

The LOS among the pediatric patients studied was 3.14 days. This duration is shorter than existing research indicating an average hospital stay of 4.76 days, either from enhancements in burn care in this region or less severe burns being addressed overall [12]. Existing research with a shorter average hospital stay of 2.5 days, compared to our 3.14 days, could be due to the presence of specialized burn units, better outpatient management, and access to resources [11]. All of these factors could allow for a quicker patient discharge and turnover, emphasizing the noticeably shorter hospital duration compared to our study. The observed pattern of overall longer LOS in the older children group (16-18 years), 5.8 days, compared to the younger children groups (0-10 years), corresponds with previous research that hypothesized recovery time and duration of hospital stay coincides with larger burn sizes [14].

Differences in LOS between age groups were assessed using a one-way ANOVA. From the stratified age groups in our study, the younger children group, ages 0-5 and 6-10 group experienced a shorter hospital duration than the older group most likely due to smaller TBSA and frequent scald burn injuries which do not require extensive care. The younger children have better recovery mechanisms than the older children group in this study due to the older children’s frequency of flame burns and presence of inhalation injuries which require greater work-up and extensive care.

The TBSA burned in the pediatric group was found to be 5.53%. There is a noticeably higher TBSA percentage in our research compared to others with a mean TBS of only 3.76% [12]. This difference could be from environmental factors in this region leading to the increased use of open flames and unsafe practices. Additionally, the burn types seen in the older group population usually arise from flame burns, indicating a large TBSA exposed compared to scalds.

A one-way ANOVA for TBSA showed significant differences in age groups in our study. Among our pediatric population, the 0-5-year and 6-10-year groups were the most populous in our study and also experienced the lowest TBSA burn. The low TBSA could be due to the predominance of scald burns in this population, the limited mobility of this group associated with smaller isolated burns, and the presence of adult supervision, unlike the older group. As the age increases among the distinct groups, there is less supervision, more independence, and greater risk-taking behaviors which results in a higher TBSA.

The region of Appalachia comes with its own unique socio-economic setbacks, such as geographically isolated populations with poor health status and the presence of chronic diseases that combine and contribute to disparities that are difficult to overcome [3]. Unfortunately, Appalachia’s rural communities struggle to obtain proper resources and burn care facilities that urban communities are equipped with. Due to the lack of burn facilities and resources, these regions are often faced with improper treatment and overall worsened outcomes and recovery [4]. Current studies believe prevention programs and education initiatives directed toward safety, particularly in regions like Appalachia, can hopefully reduce the incidence of burns in the pediatric population. Equipping the rural community with telemedicine services will also allow an isolated population to have access to expert burn clinicians for remote care and consultations to restore the existing healthcare gap [15]. Overcoming regional healthcare disparities is essential for improving outcomes in rural populations. Additionally, needing to address regional healthcare disparities, including the establishment of specialized burn units, is vital to improving burn outcomes.

The results reinforce the need for community-based strategies based on age, specifically focusing on education about scald risks in the younger children groups and encouraging flame education for the older children groups [6,16]. Previous research emphasizes the need for burn prevention and educational programs, recognizing the impact of preventing pediatric burn injuries [17]. Our study draws attention toward the pediatric population in hopes of providing a strategy to supplement clinical care and prevention efforts specific to this vulnerable population.

Future studies in the pediatric burn population could be advantageous in exploring long-term outcomes in the region, recovery, psychological functioning after the burn, and potential challenges of living in Appalachia [18]. To reduce disparities and improve overall outcomes in this population, issues including limited healthcare access and expanding burn center services in the region are vital for the community and need to be addressed.

There are a few limitations to this study. This study’s design and reliance on data from a single burn center, such as Cabell Huntington Hospital, limit its generalizability to other Appalachian regions or broader populations. In addition, this study exclusively focused on the pediatric population (0-18 years) who received care in Huntington, WV, so this information is not generalizable toward the adult population, and further research is needed. Another limitation of our study is from using one health system to gather information which allows for the possibility of incomplete data, which would ultimately compromise overall external reliability and validity.

## Conclusions

In conclusion, our study highlights observable pediatric burn patterns among four distinct age groups, “age 0-5”, “age 6-10”, “age 11-15”, and “age 16-18” in the Appalachian region. The younger children group was at an increased risk of preventable household scald burns, compared to the adolescents’ increased susceptibility to flame burns. The older children group also experienced longer hospital stays and more body surface area burns on average compared to the younger children group. There should be a requirement for specific prevention strategies based on age and resources for the Appalachian community to hopefully lower the incidence of unintentional burn injuries. Future research and studies on pediatric burns should work toward developing proper and adequate care for rural communities in addition to examining the disparities and the effect of comorbidities on burn patients in areas such as Appalachia.
